# Retraction Note: IGF-1 protects tubular epithelial cells during injury via activation of ERK/MAPK signaling pathway

**DOI:** 10.1038/s41598-026-67547-1

**Published:** 2026-09-07

**Authors:** Zengbin Wu, Yang Yu, Lei Niu, Aihua Fei, Shuming Pan

**Affiliations:** Department of Emergency, Xinhua Hospital, Shanghai Jiaotong University Medical College, Shanghai, 200092 China

Retraction of: *Scientific Reports* https://doi.org/10.1038/srep28066, published online 15 June 2016

The Editors have Retracted this Article.

Following publication, concerns were raised regarding similarities between images in this Article and images published previously elsewhere ^1,2^. Specifically, Figure 1B, UUO+IGF-1 appears to be similar to Figure 2d of^1^ and Figure 3A, alpha-tubulin appears to be similar to Figure 5A, ß-actin of^2^. The Authors were contacted about this but did not respond.

None of the Authors responded to any correspondence from the Editors regarding this retraction.

## References

[1] Xu, C., Wang, W., Xu, M. & Zhang, J. Asiatic acid ameliorates tubulointerstitial fibrosis in mice with ureteral obstruction. *Exp. Ther. Med.* **6**, 731–736 (2013) (**Spandidos Publications: Experimental and Therapeutic Medicine**). 24137256 10.3892/etm.2013.1197 PMC3786849

[2] Li, Z. et al. SIRT2 inhibits non-small cell lung cancer cell growth through impairing Skp2-mediated p27 degradation. *Oncotarget* **7**(14), 18927–18939 (2016). 26942878 10.18632/oncotarget.7816 PMC4951341

